# Development of a quality metric to measure bystander cardiopulmonary resuscitation in out of hospital cardiac arrest: A modified Delphi study

**DOI:** 10.1016/j.ijnsa.2026.100714

**Published:** 2026-10-17

**Authors:** Melinda (Dolly) McPherson, Sarah McLachlan, Hilary Bungay, Thomas R Keeble, Gareth Grier, Adam Carr

**Affiliations:** a Anglia Ruskin University, Chelmsford, UK; b Essex & Herts Air Ambulance, Essex, UK; c Essex Cardiothoracic Centre, Essex, UK

**Keywords:** Out-of-hospital cardiac arrest, Cardiopulmonary resuscitation, Delphi technique, Resuscitation

## Abstract

**Background:**

Out-of-hospital cardiac arrest remains a significant global public health challenge, with survival heavily dependent on early and high-quality bystander cardiopulmonary resuscitation. Current data collection frameworks lack standardised tools to assess the quality of bystander cardiopulmonary resuscitation.

**Objective:**

This study aimed to develop a preliminary consensus-based quality metric for use in both research and clinical practice.

**Setting:**

This study was conducted in the United Kingdom with international representation within the Delphi panel.

**Participants:**

A total of 104 participants registered, with 62, 48, and 18 completing rounds one to three, respectively in the Delphi panel. The panel consisted of Doctors, advanced practitioners, Paramedics, Nurses and academics.

**Methods:**

A modified Delphi method was employed to identify and define quality indicators. Criteria were developed from a literature review, stakeholder input, and panel consensus. A diverse expert panel, including clinicians, researchers, educators, and informed laypersons, participated in three iterative rounds (two online surveys and a final virtual consensus meeting). Consensus was defined as ≥75% agreement on a 1–5 Likert scale.

**Results:**

Eleven criteria achieved consensus for inclusion, encompassing core mechanical parameters (e.g., chest compression rate, depth, recoil) and contextual factors (e.g., dispatcher instruction, time from recognition to emergency call, and automated external defibrillator use). All criteria were agreed to be scored dichotomously. Two composite indicators were created to streamline overlapping elements.

**Conclusion:**

The provisional metric reflects both established guidelines and evolving evidence on factors influencing bystander cardiopulmonary resuscitation effectiveness. The inclusion of time and system-based criteria acknowledges the broader context in which cardiopulmonary resuscitation is delivered. Once validated in future studies this metric has potential for practical relevance for feedback tools, service evaluation, and research standardisation. This study was not registered.

**Highlights**

**What is already known**

• Out-of-hospital cardiac arrest is a major global health concern where survival is heavily dependent on early, high-quality bystander cardiopulmonary resuscitation
• While core mechanical parameters like compression rate and depth are widely endorsed in guidelines, current data collection frameworks (such as the Utstein reporting template) lack standardised, validated tools to specifically assess the quality of bystander-delivered cardiopulmonary resuscitation in out-of-hospital cardiac arrest.

**What this paper adds**

• This study introduces a novel, but as yet unvalidated quality metric consisting of 11 criteria including mechanical parameters and contextual factors developed through a formal modified Delphi process.
• The paper introduces a simplified, dichotomous scoring system and composite indicators designed for clinical usability and ease of real-world application.

## 1 Introduction

Out-of-hospital cardiac arrest is a major global public health concern, with only 8.8% of adults surviving to hospital discharge globally (Grasner et al., 2024; PAROS., 2025). The 'chain of survival' outlines essential steps to improve outcomes, with early cardiopulmonary resuscitation being critical; each minute without cardiopulmonary resuscitation reduces survival chances by 7–10% (Kragholm et al.*,* 2017; Giugni et al.*,* 2018). Bystander cardiopulmonary resuscitation has been consistently linked to improved survival rates and neurological outcomes, making it a key determinant of out-of-hospital cardiac arrest prognosis (Yu et al., 2020; Simmons et al., 2023; Semeraro et al., 2025). Despite its importance, little is known about the quality of bystander cardiopulmonary resuscitation (Gyllenborg et al.*,* 2017), and no consensus-based or validated metric currently exists for assessing cardiopulmonary resuscitation quality delivered by bystanders.

The Utstein reporting template for out-of-hospital cardiac arrest, is an internationally used framework designed to track, collect, and compare registry data for cardiac arrest and resuscitation outcomes. It includes bystander cardiopulmonary resuscitation as a recordable outcome but does not assess its quality (Bray et al.*,* 2024), leading most research to rely on the presence or absence of bystander cardiopulmonary resuscitation without reference to quality. While the Utstein template provides important global epidemiological categorisation, its failure to evaluate variations in quality limits insight into bystander efficacy. A new metric is necessary to fill that gap. Scoring systems currently found in the literature range from highly technical, device-dependent metrics developed in controlled environments (Riyapan et al.*,* 2019; Park et al.*,* 2020; Lancaster, Stilley and Franke, 2021) to observational tools developed for an individual study (Linderoth et al.*,* 2021; Wik, Steen and Bircher, 1994; Van Hoeyweghen et al.*,* 1993). There is however a paucity of observational tools suited to real-time application, with none developed specifically for use outside of their intended study or validated for widespread application. Given the limited accessibility of real-time feedback technology in the context of bystander cardiopulmonary resuscitation, assessment of cardiopulmonary resuscitation quality is commonly conducted by pre-hospital clinicians and dispatch using subjective application of guidelines (Hawkins et al.*,* 2024; Faddy et al.*,* 2026), although this is not standardised in application or reporting.

Guidelines by the International Liaison Committee on Resuscitation and the American Heart Association define quality in terms of the mechanical parameters of chest compressions. These include ‘compressions in the correct position’, ‘compression depth 5–6cm’, ‘compression rate 100–120′ and ‘ensuring full release while minimising interruptions.’(Panchal et al.*,* 2020; Smyth et al.*,* 2025) However, there has been recognition since the International Liaison Committee on Resuscitation 2015 treatment recommendations that other factors might also impact on quality (for example the position of the rescuer or the patient) thus acknowledging the complex relationship between environmental and mechanical factors in the provision of cardiopulmonary resuscitation (Perkins et al.*,* 2015). Such acknowledgement of other factors contributing to quality in resuscitation guidelines, shows a significant step towards assessing the wider aspects of quality. Subsequent guidelines have increasingly included analysis of the underpinning evidence and review of the ongoing relevance of factors such as automated external defibrillator use, the surface where the patient is found, dispatch processes and more recently artificial intelligence and technological advancements (Nolan, Jerry P. et al.*,* 2020; Olasveengen et al.*,* 2021; Berg et al.*,* 2024; Kleinman et al.*,* 2025). However, these factors are typically evaluated as independent variables in relationship to survival outcomes and there has been no consideration of how these might work together as a combined measure of quality.

The need for a comprehensive and standardised bystander cardiopulmonary resuscitation metric is further validated by the introduction of video-assisted dispatch technologies into emergency medical services (Lin et al.*,* 2018; Pan et al.*,* 2022; Linderoth et al.*,* 2021). As dispatch processes evolve from audio-only instruction to visual feedback, the capacity to observe bystander performance in real-time offers the potential for immediate quality improvement alongside real time quality data recording.

The metric developed through the research presented here is tailored for multi-disciplinary application across the resuscitation journey. The target end-users for it include pre-hospital clinicians, out-of-hospital cardiac arrest researchers, cardiopulmonary resuscitation educators, and emergency medical dispatchers. It is anticipated that the quality metric will initially be applied using video assisted dispatch. Paramedics working in dispatch roles (such as specialised cardiac arrest desks (EEAST, 2025) might grade the quality of cardiopulmonary resuscitation during an emergency call, record the result in the patient record for ongoing prognosis and future research opportunities. As the metric is developed further during validation studies it is anticipated that its use could be adapted for use by pre-hospital clinicians arriving on the scene of an out-of-hospital cardiac arrest where bystander cardiopulmonary resuscitation is ongoing.

A fully validated quality metric for bystander cardiopulmonary resuscitation could enhance service improvement, research design, and real-time guidance. Practically, providing a benchmark to evaluate intervention effectiveness and establishing specific criteria for call-handler feedback. By standardising cardiopulmonary resuscitation quality into distinct tiers, researchers can control for quality as a confounder when evaluating post-arrest interventions. Given its alignment with global basic life support standards, the metric holds strong potential for international application.

This study aims to develop a preliminary consensus quality metric for bystander cardiopulmonary resuscitation in out-of-hospital cardiac arrest. This metric will require further reliability and validation studies and as such is reported as provisional.

## 2 Methods

A three-round modified Delphi approach was used, preceded by a literature review to inform the initial criteria to be considered by the Delphi panel. The study has been reported in accordance with the Conducting and REporting DElphi Studies reporting guideline (Hasson, Keeney and Mckenna, 2025; Jünger et al.*,* 2017).

## 3 Literature review methods

### 3.1 Search design

To identify relevant criteria of quality to be presented to the modified Delphi panel, a literature review was conducted in April 2025. Databases included; Cumulative Index to Nursing and Allied Health Literature, Embase, Medline, PubMed, Science Direct, Scopus, Cochrane and Wiley Online Library. Additionally, resuscitation guidelines were sourced from Resuscitation Council United Kingdom, American Heart Association, National Institute for Health and Care Excellence, European Resuscitation Council and the International Liaison Committee on Resuscitation.

The research question that guided the review was: *‘How does the literature define and measure quality for bystander cardiopulmonary resuscitation in out-of-hospital cardiac arrest?’*

Free-text terms and relevant Medical Subject Headings combined with Boolean operators: ‘AND’ and ‘OR’ were used as below:

("bystander CPR" OR "bystander cardiopulmonary resuscitation" OR "bystander resuscitation" OR "bystander intervention" OR "early resuscitation" OR "community resuscitation" OR "first aid" OR "lay responder" OR "lay rescuer*" OR "layperson*" OR "public")

AND

("out of hospital cardiac arrest" OR "OHCA" OR "out of hospital sudden cardiac arrest" OR "OHSCA" OR "pre-hospital cardiac arrest" OR "public cardiac arrest" OR "community cardiac arrest")

AND

("CPR quality" OR "high-quality CPR" OR "resuscitation quality" OR "quality" OR "effectiveness" OR "efficacy" OR "success" OR "patient outcomes" OR "patient survival" OR "survival rate*" OR "survival at 30 days" OR "mortality" OR "return of spontaneous circulation" OR "ROSC" OR "neurological outcome*" OR "cerebral performance")

This corresponded with the ‘Population, Intervention, Comparison, Outcome(s)’ framework:

Population – Adult patients with out-of-hospital cardiac arrest
Intervention – Bystander cardiopulmonary resuscitation
Comparison – N/A
Outcome(s) – Return of spontaneous circulation, Survival to discharge, Survival at 30 days, favourable neurological outcome (Cerebral Performance Category 1 or 2)

Studies were eligible for inclusion if they evaluated bystander cardiopulmonary resuscitation quality or its effect on clinical outcomes in adult (18 years) out-of-hospital cardiac arrest. Primary research, systematic reviews, and meta-analyses published in English within the last 10 years were included. In-hospital arrests, paediatric populations (<18 years), non-empirical publications (editorials, opinion pieces, single case reports), and non-English texts were excluded.

### 3.2 Screening and selection

Data extraction was conducted by the primary researcher (DM) using an excel extraction form to capture study characteristics (author, year, country, study design, and sample size), bystander cardiopulmonary resuscitation quality parameters, provider characteristics, and clinical outcomes (return of spontaneous circulation, survival, and neurological status). Where comprehensive International Liaison Committee on Resuscitation systematic reviews existed for specific categories, these syntheses were adopted as established baselines, with data extraction and synthesis focused on primary studies published after the relevant International Liaison Committee on Resuscitation search cut-off dates to capture contemporary evidence.

### 3.3 Literature review criteria development

Included articles were divided into ‘themes’ or ‘categories’ of bystander cardiopulmonary resuscitation quality (Compression parameters, automated external defibrillator, provider characteristics, patient positioning, feedback devices, relationship to the patient, prior training, number or providers and dispatch instruction). These extracted themes were reviewed by the research team to identify criteria relevant to metric development. Criteria with limited evidence were intentionally retained to prevent premature exclusion based on researcher bias. The criteria associated with quality of bystander cardiopulmonary resuscitation identified through the literature review were then put to the modified Delphi expert panel to consider

## 4 Modified delphi methods

Two online survey rounds followed by a virtual face to face meeting were used during this modified Delphi study. The modified approach was defined by the addition of a virtual final face to face round, (Alshibani et al.*,* 2020; Ramage, Mclachlan and Williams, 2023; Schneider et al.*,* 2016) and use of pre-selected criteria from a literature search (Hasson, Keeney and Mckenna, 2025). The modified Delphi approach was selected to streamline the process, balance the strengths and weaknesses of the evidence base and allow for active, real-time debate to resolve complex, low consensus items that traditional sequential surveys often fail to manage (Hasson, Keeney and Mckenna, 2025, 2000). Allowing panellists to suggest potential quality criteria during Round 1 sought to mitigate the risks of selection bias from the evidence synthesis. The Delphi technique is a recognised method for synthesising knowledge when there is limited empirical evidence available (Jünger et al.*,* 2017; Khodyakov, 2023) and is commonly used in the development of research priorities and healthcare quality indicators (Niederberger et al.*,* 2024; Shang, 2023). While there is no universal definition of the Delphi method (Shang, 2023), it is characterised by anonymity, iteration, controlled feedback and statistical group response (Khodyakov, 2023). The goal of the Delphi process is to reach consensus on a defined topic or topics (Shang, 2023; Barrios et al.*,* 2020).

### 4.1 Recruitment and panel selection

Participants were recruited via posters distributed across relevant organisations (including national pre-hospital research networks, the Resuscitation Council UK, the European Resuscitation Council, and the International Liaison Committee on Resuscitation); social media dissemination on X, Facebook, and Instagram; word of mouth; and direct invitations to out-of-hospital cardiac arrest researchers identified through the literature review. This study sought to recruit a range of experts in recognition that the knowledge of bystander cardiopulmonary resuscitation can be different according to personal and professional experiences. Minimal inclusion criteria included persons with clinical or practical experience of providing cardiopulmonary resuscitation but those with additional educational or academic experience were explicitly sought. Participants were excluded if they had provided cardiopulmonary resuscitation as a bystander rather than in a clinical context, if they were under 18, or if they did not have a history of cardiopulmonary resuscitation or dispatch training. In acknowledgement of the differences between clinical, academic and lived experiences, and to help facilitate a representative heterogeneous panel, an expert for this Delphi study was defined across three levels:

1. *Experts (Registered professionals with academic influence):* Including clinically qualified, those with resuscitation science publications or policy and guideline-level involvement. This ensured representation across the entire chain of care, including emergency dispatch, field response, and academic policy, thus defending against biases from single-specialty panels.
2. *Specialists (Registered professionals and cardiopulmonary resuscitation instructors):* Clinically qualified, with cardiopulmonary resuscitation provision or teaching experience. Therefore, those with ongoing clinical experience of cardiopulmonary resuscitation and knowledge of the practical application of cardiopulmonary resuscitation guidelines were represented.
3. *Informed individuals (Cardiopulmonary resuscitation experience only):* Those with experience in cardiopulmonary resuscitation (e.g., dispatchers, community first responders, emergency medical technicians or assistants). This group represented those who are not registered clinicians but might use the metric.

### 4.2 Study design

Participants who registered via an online platform were invited to participate in three rounds. The first two rounds were conducted in an online survey format using Jisc online surveys software V3 (JISC, 2025). Participants evaluated the suitability of criteria identified from the literature review by rating each item’s perceived importance for improving patient outcomes on a 5-point Likert scale (1 = not important, 5 = very important). In instances where a panel member did not feel that they could make an informed decision, an ‘Unable to comment’ response option was provided. This was given to ensure that panel members were not forced to make an uninformed decision. Criteria were included in the metric if they reached the 75% agreement threshold for scores of 4–5 on the Likert 5-point scale or excluded if they reached 75% agreement threshold for scores of 1–2. Those that did not reach consensus for inclusion or exclusion were progressed to the next round. This was decided a-priori and aligns with Delphi best practise recommendations (Schneider et al.*,* 2016; Barrios et al.*,* 2020). Items failing to reach consensus by the third round were excluded (Diamond et al.*,* 2013).

### 4.3 Delphi rounds

Round 1 presented the panel with the criteria developed through literature review and patient and public involvement and engagement. The focus was on decisions regarding the suitability of these criteria to be included in the final metric. In addition to Likert scoring, participants were given the option to provide free text justifications for their responses. During this round, participants were also able to suggest additional criteria to be reviewed in round 2.

Between Round 1 and Round 2 individualised feedback was provided. As controlled feedback is integral to the Delphi technique (Barrios et al.*,* 2020; Hasson, Keeney and Mckenna, 2025) participants were provided with their individual Round 1 score presented alongside the group median, interquartile range, and a breakdown of response distributions categorised by Likert score (1–2, 3, or 4–5). Additionally, an anonymised and summarised overview of the free text feedback relating to each criterion was provided. To ensure that this process did not introduce bias the summarised feedback was reviewed prior to distribution by the Patient and Public Involvement and Engagement team and academic researchers.

Round 2 presented the panel with newly suggested criteria and those that had not reached consensus in Round 1. In addition, participants were asked to consider how already included criteria (from Round 1) might be measured. This step accounted for differences in criterion complexity: while certain factors contribute to quality through their presence (and can be evaluated dichotomously as Yes/No), others, such as chest compression rate, where current guidelines recommend 100–120 compressions per minute (Olasveengen et al.*,* 2021), may require more specific categorisation, as performance outside these parameters could represent lower-quality cardiopulmonary resuscitation. Consequently, two possible approaches were presented to the panel to vote on.

1. *Scoring using sub-categories:* for example, rating adherence to target compression rate as <25%, 25–50%, 50–75%, or >75% of the resuscitation duration; or
2. *Dichotomous scoring:* indicating whether the criterion was present or not, based on a pre agreed proportion of the total resuscitation time.

Individualised feedback including group averages and summarised free text responses was again provided between Round 2 and Round 3, incorporating the results from both the criteria selection voting and scoring methods voting.

The final round (Round 3) took place via a virtual video meeting facilitated by two lead researchers *(DM, SM),* where participants engaged in structured group discussions to further refine and finalise the metric. Following stages of discussions, participants voted anonymously on final criteria using the online voting software Slido (Cisco systems, 2025). During this round the panel were asked to review final criteria that had not reached consensus, vote on scoring methods for included criteria and finally consider the feasibility and usability of the metric. In Round 3, to ensure a definitive outcome, decisions regarding how to measure already agreed-upon criteria defaulted to a simple majority vote if the primary consensus threshold was not achieved. However, inclusion and exclusion of criteria remained bound by the 75% consensus threshold, with non-consensus criteria excluded by default at the end of Round 3 (Fig. 1).

**Fig. 1:**
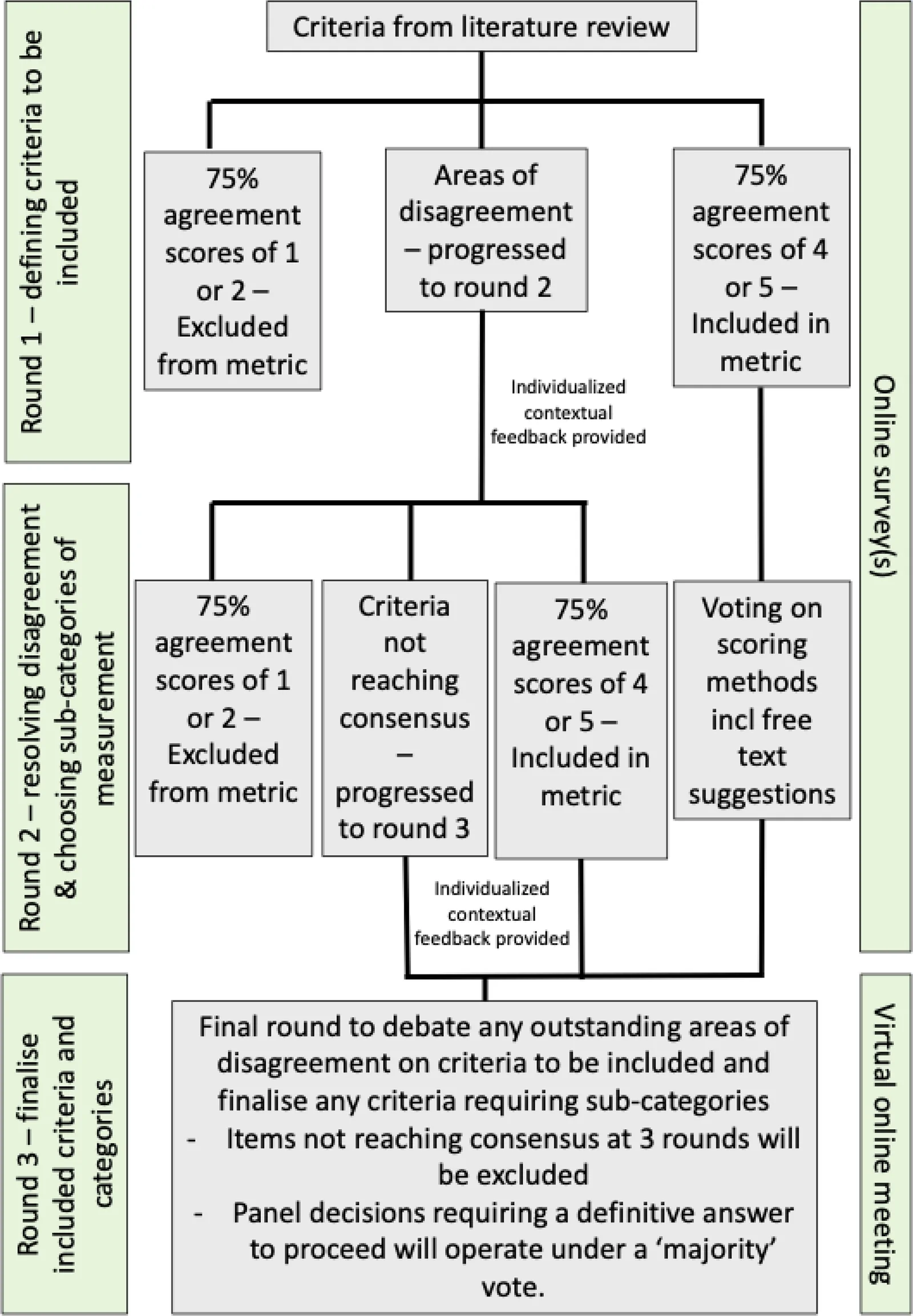
Delphi round process chart.

## 5 Patient and public involvement and engagement

A patient and public involvement and engagement advisory group, including cardiac arrest survivors, previous bystander cardiopulmonary resuscitation providers, and public members, guided survey design, panel recruitment, and results interpretation. In alignment with the Core Outcomes in Cardiac Arrest statement (Haywood et al.*,* 2026) Patient and public involvement in research is increasingly recognised as a cornerstone of robust ethical and effective research (National Institute for Health and Care Research (NIHR), 2026; Li, Richards and Primeau, 2025). It seeks to engage patients and public to understand their experiences, empower them to influence the research journey and ensure that research is conducted with the patient at the core of the research values and aims (Li, Richards and Primeau, 2025; National Institute for Health and Care Research (NIHR), 2026). Public members were defined as non-healthcare professionals who represent the community who have, or who are likely to perform bystander interventions. The inclusion of this group, particularly those who have survived out-of-hospital cardiac arrest and those who have provided bystander cardiopulmonary resuscitation, offered invaluable perspectives. The group were asked to contribute towards; the overall concept of the study design; review of participant information sheets, the notion of ‘scoring’ quality in bystander cardiopulmonary resuscitation; reviews of group feedback; and contributed to the inclusion of two additional criteria in the Delphi process

## 6 Ethical considerations

The research obtained ethical approval from Anglia Ruskin University Faculty of Health, Medicine and Social Care School Research Ethics Panel, approval number ETH2425–4566. All participant data were pseudonymised during the active survey phases and fully anonymised upon completion of Round 3, Participants were anonymous to each other until the virtual meeting in the final round, however all voting remained anonymous. To safeguard against the influence of individual professional biases, a diverse panel of experts was purposefully recruited to ensure a balanced distribution of perspectives. Furthermore, complete participant anonymity was maintained throughout the iterative voting processes. This design aimed to protect panel members from peer pressure or fear of professional exposure. Finally, all participants received a comprehensive participant information sheet which detailed the study's aims, methods, and details of what would be expected of them during participation in the research. Subsequently, informed consent (with express right to withdraw at any point) was obtained from every individual who agreed to participate, signifying their voluntary agreement.

## 7 Results

### 7.1 Literature review results

A total of 7920 records were initially identified, following screening and deduplication, 69 primary articles and systemic reviews were included. Synthesis identified eight core domains of bystander cardiopulmonary resuscitation quality: chest compression parameters, Automated external defibrillator, prior training, provider characteristics, bystander–patient relationship, patient positioning, feedback devices, and number of rescuers. These domains directly informed the candidate criteria for the subsequent Delphi panel. A comprehensive summary of the results is available in Supplementary File 1.

### 7.2 Modified Delphi results

A total of 104 participants initially registered for the Delphi study; of these 62 completed Round 1, 48 completed Round 2, and 18 attended the final virtual round (Table 1). Participants were mainly from England (79%), with others from Wales, Scotland, Australasia, Europe, and Canada. Mean length of clinical experience in years was 18.81 (Round 1), 19.27 (Round 2), and 20.39 years (Round 3); mean length of research experience was 7.17, 9.30, and 9.94 years respectively. Participants held a wealth of experience and credentials across clinical, educational and academic fields (Fig. 2 and Table 1).

**Table 1:** Expert panel clinical and academic credentials by round.

Professional registration	Round 1	Round 2	Round 3
Doctors	28 (45%)	22 (46%)	8 (44%)
Advanced practitioners (Nurse or Paramedic)	12 (19%)	8 (17%)	3 (17%)
Paramedic	11 (18%)	9 (19%)	5 (28%)
Nurse	4 (7%)	5 (10%)	2 (11%)
No clinical registration	7 (11%)	4 (8%)	0 (0%)
**Clinical and academic experience**⁎			
Directly delivered cardiopulmonary resuscitation in clinical Settings	62 (100%)	48 (100%)	18 (100%)
Certified advanced life support instructors	43 (70%)	33 (69%)	14 (77%)
Active contributors to cardiopulmonary resuscitation advocacy, dispatch, or support networks (*panellists with explicit expertise in the early links of the chain of survival, encompassing emergency services dispatchers, public health policy leaders, Community First Responder coordinators, and clinical leads who actively design or deliver public-facing cardiopulmonary resuscitation advocacy campaigns, telephone-assisted triage protocols, and community defibrillator initiatives)*	32 (52%)	24 (50%)	9 (50%)
Published peer-reviewed resuscitation literature	22 (35%)	18 (37%)	8 (44%)
Authored or contributed to national/international guidelines *(International Liaison Committee on Resuscitation, European Resuscitation Council, Resuscitation Council United Kingdom, American Heart Association)*	17 (27%)	14 (29%)	6 (33%)

⁎ Totals do not add up to 100% due to ability of the panel to select multiple credentials.

**Fig. 2:**
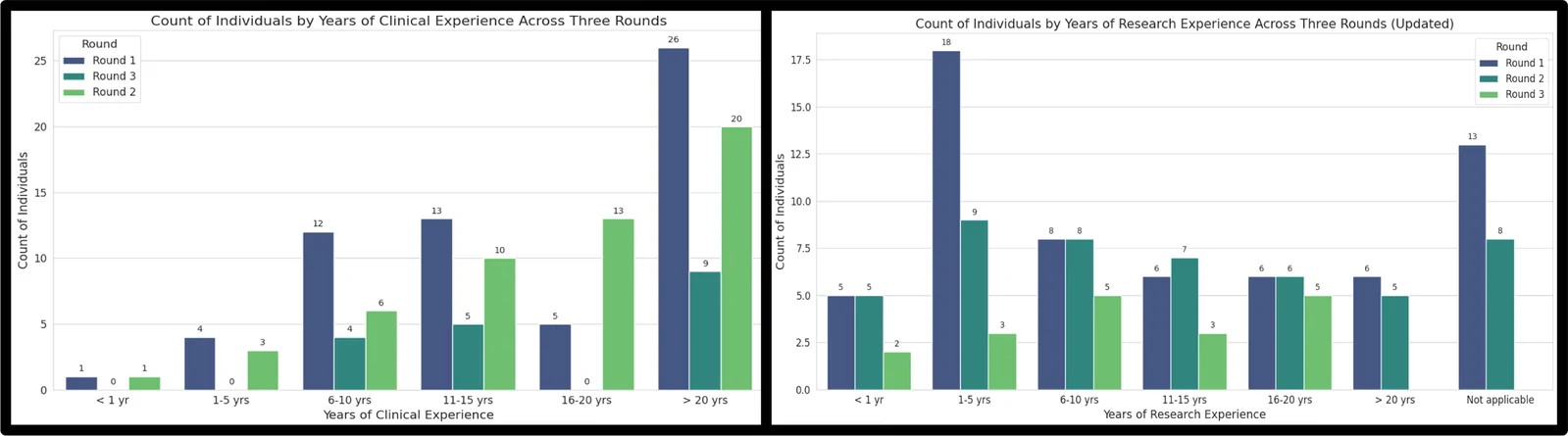
Expert panel clinical and research experience by round.

### 7.3 Round 1

In Round 1 following the literature review and Patient and Public and Involvement Engagement 16 criteria were put to the expert panel (Table 2). Consensus was reached on the inclusion of 11 of the original 16 criteria. No criteria were excluded, and six new criteria were suggested by the panel for inclusion in Round 2. (Fig. 3)

**Table 2:** Criteria presented to the Delphi panel in Round 1 and 2.

Round 1 – Criteria developed through literature review and patient and public involvement and engagement input	Round 2 - Additional criteria suggested by the expert panel
1. Chest compression – rate	1. Location of the arrest
2. Chest compression – depth	2. Defibrillator pad placement
3. Chest compression – recoil	3. Time to defibrillation
4. Chest compression – hand placement	4. Recognition of cardiac arrest
5. Chest compression – pauses	5. Phone in speaker mode
6. Chest compression – time to start	6. Duration of bystander Cardiopulmonary resuscitation
7. Time to emergency call⁎	
8. Defibrillation – shocks given	
9. Automated external defibrillator on scene	
10. Dispatcher Cardiopulmonary Resuscitation instruction	
11. Hard surface	
12. Prior training of the bystander	
13. Relationship of the bystander to the patient	
14. Number of bystanders	
15. Age of the bystander	
16. Physical characteristics of the bystander⁎	

⁎ Suggested by Patient and Public Involvement and Engagement panel

**Fig. 3:**
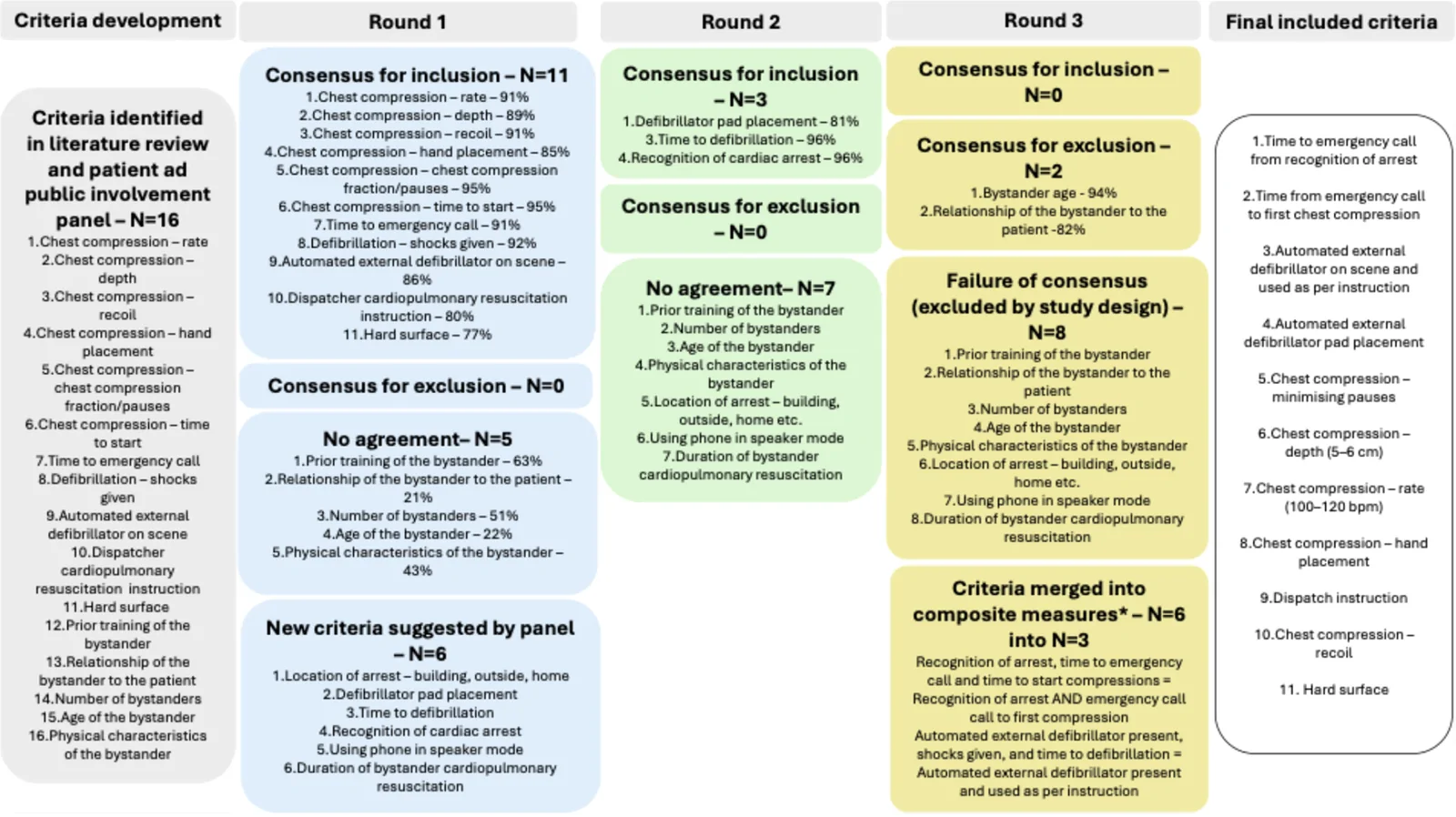
Summary of results for each of the quality criterion by round. (*Composite criteria resulted in 11 final criteria despite 14 criteria reaching consensus).

### 7.4 Round 2

The five criteria that did not reach consensus in Round 1 also failed to reach consensus in Round 2. Three of the six new criteria suggested in Round 1 by the panel reached consensus for inclusion. Nine of 11 included criteria were agreed to be scored dichotomously while two did not reach consensus for either scoring option; new criteria were not subject to voting on dichotomous or sub-categorical measurement at this stage. At the end of Round 2 fourteen criteria had reached consensus for inclusion within the metric. Eight further criteria failed to reach consensus (Fig. 3).

### 7.5 Round 3

In Round 3, of the eight remaining non-consensus criteria, two were excluded by consensus agreement of >75% on a Likert scale of 1–2. The remaining six criteria did not reach consensus so were excluded by default as per study design. (Fig. 3). Criteria that did not reach consensus were subsequently excluded in Round 3 and all had a sizeable reduction in Likert scores 4 or 5 during Round 3. Expert panel discussions on final criteria inclusion centred heavily on real-world practicality and usability. Panellists argued that poor interrater reliability and subjective interpretation would render the metric unusable. Additionally, the panel considered whether remaining criteria were already encompassed by or mitigated by included items (e.g., prior training mitigated by effective dispatcher instruction).

### 7.6 Round 3 – combining criteria

Following concerns raised by the Patient and Public Involvement and Engagement team, comments in the free text results, and research team reflection, discussions among the Delphi panel were held regarding the implications of criteria specifically related to shockable rhythms. Consideration was made as to how these might bias a quality score in favour of bystanders providing cardiopulmonary resuscitation to those presenting in ventricular fibrillation or ventricular tachycardia. The panel, by consensus (94% agreement), felt that combining some automated external defibrillator related metrics to create a single criterion inclusive of all cardiac arrest rhythms might mitigate this. When asked to vote on which criteria should be merged into a composite criterion the panel agreed by consensus that ‘automated external defibrillator present (81%),’ ‘shocks given (81%),’ and ‘time to defibrillation (75%)’ should be merged into ‘automated external defibrillator present and used as per instruction.’.

Additionally, with multiple criterion all measuring the time to commence chest compressions (Time to start chest compressions, recognition of arrest, and time to emergency call), the panel suggested that these form two composite criteria to encompass the key time points of ‘recognition of arrest to emergency call’ and ‘emergency call to first chest compression’. This decision was based on the need to establish measurable baseline data, as many out-of-hospital cardiac arrests occur unwitnessed without a verifiable time of onset. Recognition of arrest was described as either the timestamp of the person being found in arrest or the point of witnessed collapse.

### 7.7 Round 3 – criteria scoring

At the end of Round 3 all 11 included criteria (nine original and two composite) were agreed to be scored dichotomously as this was felt most practical for real world use. Dichotomous scoring was agreed by consensus for: chest compression rate (95%), chest compression depth (93%), chest compression hand placement (93%), minimal pauses in chest compressions (94%), chest compression recoil (92%), hard surface (100%), dispatch instruction (100%), defibrillator pad placement (100%) and Automated external defibrillator present and used as per instruction (92%).

Only the time-to-start metrics did not reach the consensus threshold and were therefore decided through majority voting. Regarding time metrics, the panel rejected ordinal scales in favour of a binary (yes/no) <3-minute quality threshold. A < 3-minute cutoff was selected for 'recognition of arrest to emergency call' by 56% of panellists (with 31% choosing <5 min) and for 'emergency call to first compression' by 88% of panellists (with 6% selecting <1 min or <5 min).

The panel showed a strong preference for pragmatic easy to measure scoring parameters. The panel felt that a defined percentage of time as a binary cut off would be too difficult to measure accurately. Instead opting for the use of descriptive terminology such as ‘majority of the time’ which was widely accepted among the panel (93%) as practical and usable. This precedent subsequently influenced the decisions regarding minimising pauses in chest compressions, noting that this terminology of minimal pauses is reflected in current resuscitation guidelines.

## 8 Provisional metric at the end of modified Delphi process

Following Three Delphi rounds a provisional metric was developed. This will be subject to validity and reliability testing moving forward (Terwee et al.*,* 2018) and is not yet ready for clinical use. The provisional 11-point metric developed through consensus at the end of three Delphi rounds is shown below:

1 – Recognition of arrest to time of emergency call - < 3 min
2 – Time from emergency call to first chest compression - <3 min
3 – Dispatcher instruction present – Yes/No
4 – Patient on a hard surface – Yes/No
5 – Chest compression rate between 100–120 bpm – for the majority of the time?
6 - Chest compression depth between 5–6 cm – for the majority of the time?
7 - Chest compression full recoil – for the majority of the time?
8 - Chest compression correct hand placement – for the majority of the time?
9 - Chest compression pauses – minimal?
10 – Automated external defibrillator present and used as per instruction – Yes/No
11 – Defibrillator pad placement visually correct – Yes/No

## 9 Discussion

This study developed a consensus-based metric to address the challenge of measuring bystander cardiopulmonary resuscitation quality in out-of-hospital cardiac arrest. Through a modified Delphi process, 11 criteria achieved consensus for inclusion. Agreement on dichotomous scoring with pre-determined cutoff points or descriptions resulted in a metric capable of producing an overall aggregate quality score. While currently unvalidated, this tool introduces a novel method of understanding and measuring bystander cardiopulmonary resuscitation quality in out-of-hospital cardiac arrest. To appreciate the utility of this new tool, it must be contextualised within the broader landscape of existing resuscitation assessment methods.

Historically, cardiopulmonary resuscitation evaluation tools have focused predominantly on educational outcomes and simulated skill retention rather than real-world bystander performance (Whitfield et al., 2003; Hussain et al.*,* 2025; ANZCOR, 2025). These methods are frequently supported by technology not available to out-of-hospital evaluation such as manikin-embedded software or artificial intelligence (Cortegiani et al.*,* 2017). Among the existing options, the Cardiff Assessment of Response and Evaluation (Donnelly et al.*,* 1998; Lester et al.*,* 1997; Whitfield et al., 2003) is the most closely aligned validated metric to our proposed tool. The Cardiff Assessment of Response and Evaluation is a validated assessment tool designed to evaluate individual cardiopulmonary resuscitation competency and skill retention in simulated environments using retrospective video analysis.

While both the Cardiff Assessment of Response and Evaluation tool and our new metric standardise cardiopulmonary resuscitation evaluation, they diverge significantly in developmental focus, environment, and scoring design. While both tools prioritise core features of cardiopulmonary resuscitation, specifically chest compressions and automated external defibrillator integration, they differ fundamentally in scope and purpose. Through international expert consensus, the new metric established in this study defines a comprehensive set of quality criteria specifically tailored to bystander cardiopulmonary resuscitation in the real-world situation. Conversely, the Cardiff assessment was designed primarily to evaluate trainee competence and long-term skill retention within simulated, and therefore relatively controlled, environments (Lester et al.*,* 1997; Donnelly et al.*,* 1998), using video recordings to overcome the limitations of traditional live assessment. In addition, the scoring frameworks are different; while the Cardiff Assessment of Response and Evaluation tool uses multi-categorical scoring to describe variations in performance quality (e.g., ‘opened as taught’ versus ‘visibly attempted’), the Delphi panel opted for a pragmatic, purely dichotomous scoring system for all 11 criteria to ensure feasibility and usability in real-world settings. Furthermore, while the Cardiff Assessment of Response and Evaluation tool relies on integrated manikin data for objective ventilation and compression measurements, the Delphi metric in this study uses observational data to overcome the absence of recording software in out-of-hospital cardiac arrest. Despite these differences, both frameworks share a core reliance on fundamental chest compression parameters to establish baseline validity.

The expert panel’s decision to incorporate fundamental chest compression parameters aligns with current international guidelines and clinical benchmarks (Kleinman et al.*,* 2025; Smyth et al.*,* 2025). These parameters form a necessary foundation, as not only have previous efforts to quantify cardiopulmonary resuscitation quality relied upon these parameters (Van Hoeyweghen et al.*,* 1993; Wik, Steen and Bircher, 1994; Talikowska, Tohira and Finn, 2015; Chen et al.*,* 2019), the educational infrastructure for resuscitation manikins is also almost exclusively built around these measurements (Innosonian, 2018; Cortegiani et al.*,* 2017). Including these widely acknowledged markers ensures the acceptability of the final metric to peers, and its applicability to real world use. Beyond simple inclusion however, it is important to recognise the interdependence of these factors. Efficacy in prolonged resuscitations is dictated by a complex interplay between rate and depth (Talikowska, Tohira and Finn, 2015; Cheskes et al., 2015) where an inverse relationship is commonly observed, with excessive compression rates compromising depth (Vadeboncoeur et al.*,* 2014; Sainio et al.*,* 2015). Consequently, literature increasingly advocates for the joint evaluation of these parameters rather than assessing them in isolation (Considine et al., 2020), a principle supported by allowing all chest compression criteria (rate, depth, recoil, pauses, hand position) to contribute to the aggregate score. Where this metric truly diverges from other study specific or educational tools, is its expansion into system-level and environmental variables.

Reflecting the expert panel’s commitment to a holistic approach to quality assessment, the metric integrates non-technical factors, including dispatcher guidance, surface type, and system response times. Including these variables supports a broader definition of cardiopulmonary resuscitation quality, reflecting the panel's instruction to prioritise criteria linked to improved patient outcomes. ‘Dispatcher instruction’, ‘automated external defibrillator use’, ‘hard surface’, ‘time from recognition to emergency call’, and ‘time from emergency call to first compression’ might all be considered novel contributors to bystander cardiopulmonary resuscitation quality. Integrating these criteria supports a broad definition of cardiopulmonary resuscitation quality that accounts for system and human factors. Consensus on 'dispatcher instruction' aligns with evidence identifying it as a critical enabler of bystander intervention and overall quality (Considine et al.*,* 2020; Hatakeyama et al.*,* 2019; Debaty and Segond, 2025; Takahashi et al.*,* 2025), which matches the European Resuscitation Council's recommendations for dispatcher-assisted cardiopulmonary resuscitation (Smyth et al.*,* 2025). Dispatch guidance improves technical compression quality and increases intervention rates in untrained populations (Takahashi et al.*,* 2025). Similarly, ensuring the patient is on a hard surface is biologically essential; a yielding surface, such as a mattress or sofa, dissipates rescuer force and compromises the ability to achieve the required 5–6 cm depth (Dewan et al.*,* 2024). The decision to incorporate precise time-to-start metrics (both from recognition to call, and from call to first compression) is strongly supported by the literature as a key component in the successful resuscitation of out-of -hospital cardiac arrest patients (Nguyen et al.*,* 2024; Harries and Ushakova, 2025; Moon et al.*,* 2020). This emphasis highlights the importance of minimising delay in the Chain of Survival (Deakin, 2018; Nolan, J., Soar and Eikeland, 2006), reinforcing the principle that swift intervention is vital for maximising the likelihood of survival with favourable neurological outcomes. Incorporating these real-world elements required the panel to adopt pragmatic, descriptive scoring criteria alongside strict quantitative cutoffs.

The use of descriptive phrasing like "majority of the time" and "minimal pauses" reflects a pragmatic approach to the challenges of real-world measurement and a lack of empirical evidence defining precise thresholds before outcomes deteriorate. Descriptive or subjective parameters are established in other clinical tools, such as the Modified Ashworth Scale for muscle spasticity (Harb, Margetis and Kishner, 2025) and the Barthel Index for activities of daily living (Sainsbury et al.*,* 2005). While less common in emergency medicine due to rapid decision-making demands, the panel viewed this as the most practical strategy available. Subjective scoring introduces potential variations in inter- and intra-rater reliability. However, an unmeasurable binary threshold cannot be superior to a pragmatic subjective approach. This metric will undergo formal reliability testing in future studies. This same pragmatic balance between empirical evidence and real-world feasibility informed the panel’s selection of time-based benchmarks.

While there is no empirically validated time threshold separating higher- from lower-quality bystander CPR the decision to use a 3-minute cut off was a pragmatic consensus driven threshold informed by the limited available evidence. Survival reduces by 7–10% for every minute without cardiopulmonary resuscitation (Larsen et al.*,* 1993) meaning the baseline probability of survival diminishes by 20–30% by this threshold. While Nguyen et al. (Nguyen et al.*,* 2024) demonstrated an inverse relationship between time to bystander cardiopulmonary resuscitation and survival to discharge, they noted a 9% reduction in survival when cardiopulmonary resuscitation occurred within 3 min compared to 1 min, which dropped further to a 27% reduction at 4–5 min. In this metric, a maximum of 6 min may elapse between arrest recognition and the first compression yet still score a positive quality result. Of course, overlap in these timings may also occur during a witnessed collapse, incurring a timeline of <3 min to call emergency services before the compression timer starts. Conversely, unwitnessed arrests may score a point if a bystander rapidly calls emergency services, potentially introducing a positive bias to a score despite a potentially poor clinical prognosis. These scoring nuances will be explored in future validity trials.

Aggregating dichotomous criteria into an overall score provides a significantly more nuanced assessment of quality than a binary indicator or vague categorical labels of "good" or "poor" (Van Hoeyweghen et al.*,* 1993; Wik, Steen and Bircher, 1994; Faddy et al., 2026). Ultimately, this proposed metric moves beyond simple binary indicators of cardiopulmonary resuscitation presence to offer a more nuanced, multidimensional framework for quality assessment.

### 9.1 Strengths and limitations

A strength of this research was the involvement of the patient and public involvement and engagement group in its development, and the size of the panel and their level of expertise. While this study provides valuable insights into defining bystander cardiopulmonary resuscitation quality, several limitations are acknowledged. The reliance on expert consensus, while appropriate for areas lacking empirical evidence, introduces subjectivity and potential bias in criteria selection and ranking. Despite attrition being a recognised limitation of Delphi studies with other studies reporting 0–92% rates of attrition (Hasson, Keeney and Mckenna, 2025; Shang, 2023), participant attrition across rounds in this study may have reduced the representativeness of final decisions, specifically, the substantial drop-off from 62 active participants in Round 1 down to 18 attendees in Round 3 (56% attrition) constitutes a significant attrition rate limitation that may over-represent the opinions of the highly specialised academic subgroup who completed the process. While the strength of the panel expertise may in part mitigate this limitation, it is a weakness in this study.

Furthermore, the metric exhibits a geographic bias, as 79% of the consensus panel was based within the English healthcare system. This concentrates findings within a specific institutional model and limits immediate international generalisability across varied emergency medical systems configurations.

Terminology limitations must also be acknowledged. The term "bystander" itself is increasingly criticised in resuscitation science because it carries a connotation of passivity or inactive observation, failing to capture the proactive nature of lay individuals who step forward to perform life-saving interventions. Using this terminology within the Delphi study, despite its common industry use could have biassed the panels perception of quality through underplaying the active nature of the intervention.

## 10 Conclusion

This study developed a consensus-based metric for evaluating bystander cardiopulmonary resuscitation quality, integrating guideline-endorsed mechanical components with broader contextual factors. Through three Delphi rounds, 11 criteria were identified and agreed upon, including novel inclusions such as time to first compression and dispatcher instruction. The preference for dichotomous scoring and simplified composite criteria reflects a focus on usability in real-world and research settings. While limitations exist, and recognition that this metric is not yet ready for clinical implementation without further validation studies, it represents a promising foundational tool for improving public health outcomes through structured assessment and benchmarking of bystander cardiopulmonary resuscitation quality in both clinical and research contexts.

## Funding

This work was supported by the Anglia Ruskin University and Essex and Herts Air Ambulance. Who match funded the Vice Chancellors PhD scholarship for Melinda (Dolly) McPherson. This funding provided course fees and UKRI stipend.

## AI declaration

During the preparation of this work the author used *ChatGPT (OpenAI)* to support editing and refining academic text; summarising meeting notes and generating alternative phrasings. After using this tool, the author reviewed, verified, and edited all content as required and takes full responsibility for the final content of the published article.

## Data availability statement

Data are not available for sharing. To protect participant confidentiality and honour privacy assurances given during informed consent, raw survey data containing potentially identifiable qualitative responses cannot be disclosed.

## CRediT authorship contribution statement

**Melinda (Dolly) McPherson:** Writing – review & editing, Writing – original draft, Validation, Project administration, Methodology, Investigation, Data curation, Conceptualization. **Sarah McLachlan:** Writing – review & editing, Supervision, Project administration, Methodology, Funding acquisition, Conceptualization. **Hilary Bungay:** Writing – review & editing, Supervision, Methodology, Funding acquisition. **Thomas R Keeble:** Writing – review & editing, Supervision, Funding acquisition, Conceptualization. **Gareth Grier:** Writing – review & editing, Supervision, Funding acquisition, Conceptualization. **Adam Carr:** Writing – review & editing, Supervision, Conceptualization.

## Declaration of competing interest

The authors declare the following financial interests/personal relationships which may be considered as potential competing interests: Melinda (Dolly) Mcpherson reports financial support, administrative support, and writing assistance were provided by Essex and Herts Air Ambulance. Professor Thomas Keeble reports a relationship with Essex Cardiothoracic Centre that includes: employment. If there are other authors, they declare that they have no known competing financial interests or personal relationships that could have appeared to influence the work reported in this paper.
