# Supplementary material for: Development of a quality metric to measure bystander cardiopulmonary resuscitation in out of hospital cardiac arrest: A modified Delphi study
Source: Int J Nurs Stud Adv. 2026 Oct 17;11:100714. doi: 10.1016/j.ijnsa.2026.100714 (PMC13720815; doi:10.1016/j.ijnsa.2026.100714)
Supplement: Supplementary file 1 [file mmc1.docx]

**Appendix (supplementary file)**

**Literature review results**

A total of 7,920 records were initially identified through database searches across CINAHL, Embase, Medline, PubMed, Science Direct, Scopus, Cochrane, and Wiley Online. Following the removal of duplicates (n=1,584), 6,336 records underwent title screening, resulting in the exclusion of 5,987 records. The remaining 349 articles were screened by abstract, where 240 papers were excluded based on predefined criteria.

Of the remaining 109 records, 40 of these were found to be adequately included and critically reviewed in published International Liaison Committee of Resuscitation systematic reviews and were therefore excluded in favour of inclusion of the associated systematic review instead. This yielded 69 articles for full-text review, with no further exclusions applied.

Eight themes pertinent to quality of bystander cardiopulmonary resuscitation were identified in the literature. These were grouped into ‘chest compression parameters’, ‘automated external defibrillator use’, ‘prior training’, ‘provider characteristics’, ‘bystander-patient relationship’, ‘patient positioning’, ‘feedback devices’, and ‘number of providers’. The PRISMA flowchart detailing literature review results is in Figure 2.

*Supplementary file Figure 1: Literature review PRISMA flow diagram*

Chest compression parameters as defined by rate (100–120 bpm), depth (5–6 cm), chest compression fraction >60%, full recoil, and hand position, were the most commonly studied indicators of quality [14,19,47,48] and feature in current national and international guidelines [11,16]. Despite limited statistical significance linking rates to survival outcomes in adjusted analyses, adherence to guideline ranges remains clinically accepted, especially for prolonged resuscitations. Automated external defibrillator use by bystanders was identified as vital for survival; observational studies and meta-analyses linked it with improved outcomes [49–51], especially for shockable rhythms [51], also offering prompts and rhythm analysis benefits [49,52].

Prior training in delivering cardiopulmonary resuscitation emerged as a consistent positive factor across 13 studies, improving cardiopulmonary resuscitation metrics, survival, and neurological outcomes [53–56], though skill retention and cohort heterogeneity limited generalizability. Bystander physical traits such as physical size or strength also influenced cardiopulmonary resuscitation quality but were largely mitigated by training [20,57]. Non-family bystanders were more likely to initiate cardiopulmonary resuscitation and use automated external defibrillators [58–60]; however, survival benefits often disappeared after statistical adjustment [58,59,61], suggesting the involvement of confounding factors. Environmental factors such as patient positioning and number of rescuers variably influenced cardiopulmonary resuscitation quality but with uncertain survival impact [62–65].

Overall, quality in bystander cardiopulmonary resuscitation appeared to extend beyond mechanical parameters to include training, automated external defibrillator use, bystander, and environmental factors. These findings informed the criteria presented in this Delphi study and are listed below.
